# Phillygenin, a Plant-Derived Lignan, Attenuates Renal Inflammation, Fibrosis, and Pyroptosis in a Unilateral Ureteral Obstruction Model

**DOI:** 10.3390/nu18091421

**Published:** 2026-04-30

**Authors:** Yu-Syuan Chen, Shun-Fa Yang, Huey-Liang Kuo, Haw-Ling Chuang, Chang-Mu Chen, Ssu-Chia Lin, Pei-Yu Weng, Chun-Fa Huang, Siao-Syun Guan, Shing-Hwa Liu, Cheng-Tien Wu

**Affiliations:** 1 Department of Nutrition, China Medical University, Taichung 40402, Taiwan; u108008441@cmu.edu.tw (Y.-S.C.); cora20001017@gmail.com (S.-C.L.); zxc0963511081@gmail.com (P.-Y.W.); 2 Institute of Medicine, Chung Shan Medical University, Taichung 40201, Taiwan; ysf@csmu.edu.tw; 3 Department of Medical Research, Chung Shan Medical University Hospital, Taichung 40201, Taiwan; 4 Division of Nephrology, Department of Internal Medicine, China Medical University Hospital, Taichung 40402, Taiwan; 012307@tool.caaumed.org.tw; 5 School of Medicine, College of Medicine, China Medical University, Taichung 40402, Taiwan; 6 Clinical Nutrition, China Medical University Hospital, Taichung 40402, Taiwan; 7 Department of Emergency, Taichung Tzu Chi Hospital, Buddhist Tzu Chi Medical Foundation, Taichung 427213, Taiwan; tc2050403@tzuchi.com.tw; 8 Division of Neurosurgery, Department of Surgery, College of Medicine and Hospital, National Taiwan University, Taipei 10051, Taiwan; cmchen10@ntuh.gov.tw; 9 School of Chinese Medicine, College of Chinese Medicine, China Medical University, Taichung 40402, Taiwan; cfhuang@mail.cmu.edu.tw; 10 Department of Nursing, College of Medical and Health Science, Asia University, Taichung 41354, Taiwan; 11 Institute of Nuclear Energy Research, Atomic Energy Council, Taoyuan 32546, Taiwan; ssguan@iner.gov.tw; 12 Institute of Toxicology, College of Medicine, National Taiwan University, Taipei 10051, Taiwan; 13 Department of Pediatrics, College of Medicine and Hospital, National Taiwan University, Taipei 10051, Taiwan; 14 Department of Medical Research, China Medical University Hospital, China Medical University, Taichung 40447, Taiwan

**Keywords:** chronic kidney disease, inflammation, fibrosis, phillygenin, pyroptosis, unilateral ureteral obstruction

## Abstract

Background/Objectives: Phillygenin (PHI), a natural lignan derived from *Forsythia suspensa*, has garnered attention for its potential to alleviate chronic diseases, including chronic colitis, pulmonary fibrosis, and diabetes. Chronic kidney disease (CKD) poses a global health challenge, characterized by high morbidity and mortality rates and associated with a spectrum of secondary complications. In this study, we aim to investigate the therapeutic effectiveness of PHI on CKD and also identify molecular signals by using a unilateral ureteral obstruction (UUO) mouse model and in vitro experiments. Methods: C57BL/6 mice were administered PHI at 50 mg/kg/day to assess its therapeutic effectiveness. In vitro, lipopolysaccharide (LPS) and adenosine triphosphate (ATP) were used to induce pyroptosis, also known as pyroptosis, in renal proximal tubular cells (NRK52E). Results: After PHI treatment for 14 consecutive days, the collagen deposition and extracellular matrix (ECM) accumulation, the expression of oxidative stress response proteins (catalase, superoxide dismutase 2, NADPH oxidase 4, and thioredoxin reductase 1), pro-inflammatory markers (TNF-α and Cyclooxygenase-2(COX-2), and infiltration of neutrophils and macrophages were significantly ameliorated in the UUO mice. Interestingly, the pyroptosis-related proteins (NLRP3/Caspase-1/GSDMD/IL-1β) and cell apoptotic death were also conspicuously relieved after treatment with PHI. Furthermore, PHI administration significantly attenuated the ATP/LPS-induced NF-κB/NLRP3/Caspase-1/GSDMD pyroptosis signal pathway in NRK52E cells. Conclusions: These results demonstrate, for the first time, that PHI treatment ameliorates inflammation and the related pyroptosis via inhibitory regulation of the NF-κB/NLRP3/Caspase-1/GSDMD axis, leading to attenuated renal fibrosis and progressive CKD in UUO mice and in vitro. Our findings suggest that PHI could be a nutraceutical candidate for attenuating CKD progression.

## 1. Introduction

Chronic kidney disease (CKD) has emerged as a major global health concern, characterized by increasing morbidity and mortality rates, and is frequently associated with a higher risk of co-morbidities such as diabetes mellitus, hypertension, and obesity [1]. Currently, more than 850 million individuals worldwide are affected by CKD, which imposes a substantial economic and health burden [2]. Renal fibrosis represents a key pathological hallmark of CKD progression and involves inflammatory cell infiltration, excessive extracellular matrix (ECM) accumulation, and epithelial–mesenchymal transition (EMT) of renal tubular cells. Increasing evidence suggests that oxidative stress and chronic inflammation act as critical drivers of these pathological processes [3,4,5,6]. During this process, tubular cells transform into interstitial fibroblasts, undergoing epithelial–mesenchymal transition (EMT), producing collagen and causing excessive synthesis of ECM, which contributes to renal tubular damage. Additionally, the excessive ROS production mediated by NADPH oxidase exacerbates the inflammatory events in the tubulointerstitial nephropathy and progression of CKD [7,8].

Pyroptosis, a form of inflammatory programmed cell death, has recently been recognized as an important contributor in chronic diseases such as metabolic liver diseases and pulmonary fibrosis [9,10,11]. This process is characterized by cellular swelling, membrane pore formation, and the release of pro-inflammatory cytokines, including interleukin-1β (IL-1β) and interleukin-18 (IL-18). Mechanistically, activation of the nuclear factor-κB (NF-κB) signaling pathway promotes transcription of inflammasome components such as NLR family pyrin domain containing 3 (NLRP3), leading to the assembly of the NLRP3 inflammasome and subsequent activation of caspase-1. Activated caspase-1 cleaves gasdermin D (GSDMD), generating the pore-forming GSDMD-N fragment that disrupts membrane integrity and triggers the release of inflammatory cytokines [12]. Interestingly, growth evidence indicates that activation of the NF-κB/NLRP3/caspase-1/GSDMD signaling axis plays a critical role in renal inflammation, fibrosis, and the progression of CKD [13,14].

Phillygenin (PHI) is a plant-derived lignan isolated from *Forsythia suspensa*, a medicinal plant widely used in traditional herbal preparations in East Asia. Previous studies have reported that PHI exhibits diverse pharmacological properties, including anti-inflammatory, antioxidant, and anti-fibrotic activities [15]. PHI has been shown to suppress NF-κB-mediated inflammatory signaling and reduce cytokine production in inflammatory disease models [16]. In addition, PHI treatment has demonstrated protective effects in several chronic disease conditions, including prevention of arthritis [17], against chronic colitis [18], pulmonary fibrosis [19], and metabolic disorders [20]. However, the potential protective effects of PHI against renal fibrosis and pyroptosis during CKD progression remain largely unexplored.

In this study, therefore, we aimed to investigate the protective effects of PHI on renal injury and fibrosis using a unilateral ureteral obstruction (UUO) mouse model and an in vitro renal tubular epithelial cell model. In particular, we focused on determining whether PHI could modulate pyroptosis-related signaling pathways involved in CKD progression. Our findings demonstrate that PHI administration alleviates renal fibrosis, inflammatory cell infiltration, oxidative stress, and pyroptosis-related signaling in UUO mice. Furthermore, PHI suppressed lipopolysaccharide (LPS) and adenosine triphosphate (ATP)-induced pyroptosis in NRK52E renal tubular cells through inhibitory regulation of the NF-κB/NLRP3/caspase-1/GSDMD signaling pathway. These results suggest that PHI may represent a promising plant-derived bioactive compound with potential nutraceutical value for mitigating the progression of CKD.

## 2. Materials and Methods

### 2.1. Animal Model and Phillygenin Treatment

**Figure 1:**
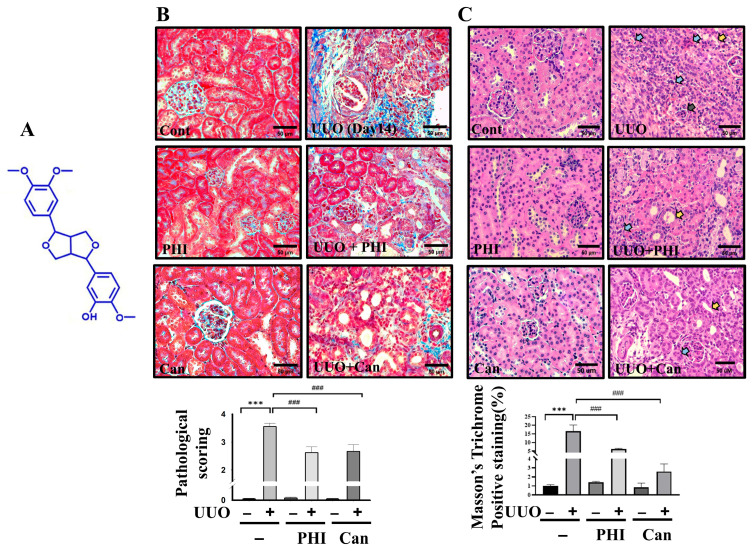
PHI attenuates renal pathological changes and collagen deposition in UUO kidneys. C57BL/6J mice were orally administered a vehicle, 25 mg/kg PHI, or 5 mg/kg Can for 14 days after UUO surgery. The chemical structure of phillygenin (PubChem Compound CID: 3083590) is shown in (**A**). Renal pathological changes were observed by H&E staining. (Blue arrow: inflammatory cells infiltration; yellow arrow: tubular dilatation; black arrow: glomerular atrophy). Renal injury scoring is presented as mean ± S.D. with 95% confidence interval (*n* = 6; Cont: 0.067, 95% CI: −0.105 to 0.238; PHI: 0.084, 95% CI: −0.205 to 0.249; Can: 0.067, 95% CI: −0.105 to 0.238; UUO: 3.330, 95% CI: 3.161 to 3.499; UUO + PHI: 2.417, 95% CI: 2.124 to 2.709; UUO + Can: 2.517, 95% CI: 2.256 to 2.777 ANOVA: *p* = 2.33 × 10^−9^; Cont vs. UUO *p* = 1.6 × 10^−7^; UUO vs. UUO + PHI *p* = 2.67 × 10^−4^; UUO vs. UUO + Can: *p* = 2.48 × 10^−5^). (**B**) Collagen deposition was assessed by Masson’s trichrome staining in (**C**). Magnification: 400×; Scale bar: 50 μm. All data are presented as mean ± S.D. with 95% confidence interval for each group (*n* = 6; Cont: 1.08, 95% CI: 0.805 to 1.138; PHI: 1.384, 95% CI: 1.015 to 1.492; Can: 0.767, 95% CI: 0.285 to 1.208; UUO: 16.14, 95% CI: 13.161 to 19.249; UUO + PHI: 5.147, 95% CI: 4.424 to 5.909; UUO + Can: 2.49, 95% CI: 2.556 to 3.277 ANOVA: *p* = 7.62 × 10^−6^; Cont vs. UUO *p* = 2.2 × 10^−4^; UUO vs. UUO + PHI: *p* = 2.67 × 10^−4^; UUO vs. UUO + Can: *p* = 2.48 × 10^−5^). *** indicates *p* ≤ 0.001 when data are compared to the Sham Control. ### indicates *p* ≤ 0.001 when data are compared to the UUO group. Cont: Sham Control; PHI: Phillygenin; Can: Candesartan.

Six-week-old male C57BL/6 mice were purchased from the National Laboratory Animal Center. (NLAC; NARLabs, Taipei, Taiwan) Animals were housed individually in standard cages under controlled environmental conditions: a temperature of 22 ± 2 °C, relative humidity maintained between 50 and 70%, and a 12 h light/dark cycle. Animal surgical operation and health care were approved by the Institutional Animal Care and Use Committee (IACUC) in the animal center of China Medical University (Taichung, Taiwan; Animal study plan: CMUIACUC-2022-335). Following one-week acclimation, mice were randomly divided into six groups: (1) control group: orally gavage the distilled water 10 mL/kg/day (*n* = 8); (2) PHI (Chem Face, Wuhan, China; PubChem Compound CID: 3083590, MW: 372.4 g/mol; structure is shown in Figure 1A) group: orally gavaged 25 mg/kg/day of PHI (*n* = 8); (3) positive control group (*n* = 6, candesartan (Can) (Sigma-Aldrich, St. Louis, MO, USA); oral gavage 5 mg/kg/day); (4) UUO surgical group; (5) UUO + PHI group (*n* = 8); (6) UUO + Can group (*n* = 6). The PHI dose used in this study was selected as a relatively low and safe dosage based on a previous report [18], which demonstrated protective effects against fibrosis. Candesartan is used as a positive control. The sample size was selected based on our previous studies and preliminary experiments to ensure adequate statistical power [21,22]. For the UUO surgical procedure, mice were briefly anesthetized with 3% isoflurane, the left ureter was ligated with 4-0 nylon thread, and the surgical site was closed with wound clips. After 14 days of post-surgery, both obstructed and contralateral kidneys were harvested for further analysis. Candesartan, an angiotensin II receptor blocker, was used as a positive control for protective effects on renal fibrosis [21,23].

### 2.2. Histopathological Examination

The 3 μm-thick paraffin-embedded renal tissue slides were prepared for both hematoxylin and eosin (H&E) and Masson’s trichrome stains. Briefly, the process commenced with the deparaffinization of the slides in xylene, followed by a rehydration sequence using progressively diluted ethanol solutions. For the H&E staining, sections were treated with hematoxylin to stain cell nuclei and eosin to stain cytoplasmic elements, facilitating the evaluation of tubular injury and other histopathological abnormalities. Renal injury was assessed across a minimum of 10 randomly selected microscopic fields by an observer blinded to the sample identities. Abnormalities were semi-quantitatively scored and graded as follows: 0 = no abnormalities; 1 ≤ 25% of tubular change; 2 = 25% to 50% of tubular change; 3 = 50% to 75% of tubular change; 4 ≤ 75% of tubular change. For Masson’s trichrome staining, sections were initially treated with Bouin’s fluid to fix tissue, followed by staining with Weigert’s iron hematoxylin working solution to highlight the nuclei. Subsequently, an aniline blue solution was applied to stain the collagen fibers. This staining sequence is particularly useful for identifying and quantifying collagen deposition and fibrosis. The extent of collagen deposition, indicated by blue staining, was quantitatively analyzed using ImageJ software (*ver.*1.53; National Institutes of Health, Bethesda, MA, USA).

### 2.3. Western Blot Analysis

The renal tissues were homogenized in RIPA buffer containing protease and phosphatase inhibitors. The lysates were centrifuged to collect the supernatant. Subsequently, the amount of total protein was quantified by using a bicinchoninic acid (BCA) kit (Bio-Rad Laboratory, Hercules, CA, USA). The western blotting procedure was performed as previously described [24]. Briefly, 20 ug of protein samples were boiled and denatured, and then loaded into 8–12% sodium dodecyl sulfate–polyacrylamide gel electrophoresis (SDS-PAGE) gels. After transferring proteins to the PVDF membranes, the membranes were blocked with 3% bovine serum albumin (BSA), incubated with primary antibodies, including fibronectin, NLRP3 (Abcam, Trumpington, Cambridge, UK); β-actin, catalase, caspase-1 p20, GAPDH, NF-κB p65 (p65), superoxide dismutase type 1 (SOD-1), tumor necrosis factor alpha (TNF-α) (Santa Cruz Biotechnology, Santa Cruz, CA, USA); α-Smooth Muscle Actin (α-SMA), Bax, caspase-3, Cyclooxygenase 2 (COX-2), E-cadherin, Gsadermin D (GSDMD), Phospho-NF-κB p65 (pp65), thioredoxin reductase-1 (TRXR1), Vimentin (Cell Signaling Technology, Danvers, MA, USA); interleukin-1 beta (IL-1β) (Affinity biosciences, Melbs, VIC, Australia); NADPH Oxidase 4 (NOX-4) (Novus biologicals, Littleton, CO, USA), overnight at 4 °C, and then incubated with secondary antibodies for 1 h. Finally, protein expression changes were detected by using an enhanced chemiluminescence substrate kit (ECL) in a digital photo-image system (Azure Biosystem, Inc., Dublin, CA, USA).

### 2.4. Immunohistochemical (IHC) Staining

Paraffin-embedded renal tissue slides with a thickness of 3 μm were prepared to assess inflammatory responses using IHC staining. Briefly, slides were deparaffinized and dehydrated with Xylene Substitute (Sub X) (Leica, Shanghai, China) and rehydrated through a graded series of ethanol solutions. For antigen retrieval, slides were immersed in Trilogy solution (Cell Marque, Rocklin, CA, USA) and boiled for 10 min. After blocking with 5% fetal bovine serum (FBS), slides were treated to remove endogenous peroxidase activity and were incubated overnight with primary antibodies, anti-Ly6g (Santa Cruz Biotechnology, Santa Cruz, CA, USA), and macrophage marker, anti-F4/80 (Abcam, Cambridge, UK), respectively. Finally, sections were chromogenically detected with 3,3′-Diaminobenzidine (DAB) (Agilent Technologies, Santa Clara, CA, USA) and hematoxylin (CIS-Bio, Gard, FR). The positively stained areas were quantified by ImageJ software (*ver.*1.53; National Institute of Health, Bethesda, MD, USA).

### 2.5. Fluorescent Terminal Deoxynucleotidyl Transferase dUTP Nick-End Labeling Assay

The paraffin-embedded renal tissue slides (3 μm thickness) were used to assess the apoptotic cells by immunofluorescence TUNEL assay. The slides were deparaffinized in SubX and rehydrated in descending concentrations of ethanol. To digest the membrane protein, proteinase K was added for 15 min, and then washed with Phosphate-buffered saline (PBS) buffer 3 times. After the preparation, slides were stained with a TUNEL assay kit (Promega, Madison, WI, USA); briefly, the slides were then incubated in the solution containing nucleotide mix and rTdT enzyme at room temperature for an hour. The counterstaining was performed by using DAPI staining (Sigma-Aldrich, St. Louis, MO, USA). The number of apoptotic cells was counted from 10 different visual fields under fluorescence microscopy with 200× magnification.

### 2.6. Cell Culture and Treatment

The normal rat kidney epithelial cell line (NRK52E) was purchased from Bioresource Collection and Research Center (Cat. No. BCRC60086; Food Industry Research and Development Institute, Hsinchu, Taiwan). The cells were cultured in High Glucose Dulbecco’s Modified Eagle Medium (DMEM/H) supplemented with 10% FBS, and 1% Penicillin/Streptomycin/Amphotericin (PSA) mixture, at 37 °C in a 5% CO_2_ incubator. For experimental treatment, cells were plated at a density of 4 × 10^5^ cells/dish in 6 cm dish, pretreated with PHI for one hour, and then followed by adding Lipopolysaccharide (10 μg/mL; LPS, Sigma-Aldrich, St. Louis, MO, USA) and Adenosine triphosphate (3 mM; ATP, Sigma-Aldrich, St. Louis, MO, USA) for 0.5–24 h, respectively. Finally, the harvested cells were analyzed by western blotting and other biochemical assays.

### 2.7. Cell Viability Assay

3-(4,5-Dimethylthiazol-2-yl)-2,5-Diphenyltetrazolium Bromide (MTT; MedChemExpress, Monmouth Junction, NJ, USA) was used to assess the cell viability after Phillygenin treatment in NRK52E cells. Briefly, cells were seeded at a density of 5 × 10^3^ cells/well in 96-well plates at different concentrations (1, 5, 20, 25, 50, 100 μM) for periods ranging from 24 to 72 h. Subsequently, the culture medium was removed, and then 0.5 mg/mL MTT solution was incubated with the treated cells for 2 h. Finally, the purple crystals were dissolved in DMSO, and the optical density (OD) was measured by using an ELISA reader (Thermo Fisher Scientific, Waltham, MA, USA).

### 2.8. Statistical Analysis

Data are presented as the mean ± standard error (S.D.) with 95% confidence interval. Statistical analysis and graphics were performed using GraphPad Prism (version 8.0; GraphPad Software, Boston, MA, USA). A one-way analysis of variance (ANOVA) followed by Tukey’s post hoc test was employed to analyze differences across multiple groups in the study. The *p*-value < 0.05 was considered statistically significant.

## 3. Results

### 3.1. PHI Treatment Attenuates Renal Fibrosis and Renal Pathological Changes in UUO Kidneys

**Figure 2:**
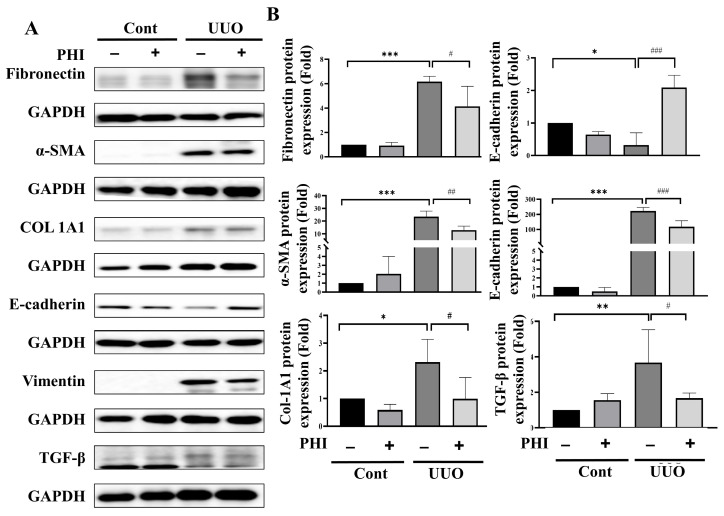
PHI attenuates protein expression of renal fibrosis and EMT markers in UUO kidneys. Protein expression levels of fibrosis-related markers were analyzed by Western blotting. Representative Western blot images of fibronectin, α-SMA, collagen, E-cadherin, Vimentin, and TGF-β. (**A**) Quantification of protein levels is shown in (**B**). Data are presented as mean ± S.D with 95% confidence interval (*n* = 6). * indicates *p* < 0.05, ** indicates *p* < 0.01, *** indicates *p* ≤ 0.001 when data are compared to the Sham Control group. # indicates *p* < 0.05, ## indicates *p* < 0.01, ### indicates *p* ≤ 0.001 when data are compared to the UUO group. Cont: Sham Control; PHI: Phillygenin.

Renal fibrosis is a critical hallmark of end-stage chronic kidney disease (ESRD), and is characterized by tubulointerstitial fibrosis and glomerulosclerosis, culminating in the deterioration of renal function [6,25]. To confirm the therapeutic efficacy of PHI treatment in UUO mice, renal pathological alterations and collagen deposition were examined using H&E and Masson’s trichrome staining, respectively. As shown in Figure 1A, the pathological abnormalities, including severe tubular dilation, glomerular atrophy, and inflammatory cell infiltration, were observed in the UUO surgical group. Conversely, these pathological changes were significantly alleviated in PHI treatment of the UUO group and the candesartan groups, which demonstrated a similar therapeutic potential as Can (positive control) (Figure 1B). Further analysis using Masson’s trichrome staining indicated excessive collagen deposition in the kidneys of UUO mice, another feature of renal fibrosis [26]. As shown in Figure 1C, collagen deposition was significantly elevated in UUO mice, which was significantly attenuated following PHI or candesartan administration, as shown by Masson’s trichrome staining. Furthermore, accumulation of ECM and epithelial–mesenchymal transition (EMT) markers has been considered to contribute to renal fibrosis and subsequent CKD [27,28,29]. Next, the protein expression of fibrosis markers, including fibronectin, α-Smooth Muscle Actin (α-SMA), collagen [30], and EMT markers, including E-cadherin, Vimentin, and TGF-β [31,32], was detected. As shown in Figure 2, these fibrotic-related proteins were significantly increased, while E-cadherin was reduced, in the UUO group. Interestingly, these abnormal protein expression patterns were markedly attenuated by PHI treatment. These results suggested that PHI treatment possesses a therapeutic benefit in ameliorating fibrosis and pathological changes in UUO kidneys.

### 3.2. PHI Treatment Alleviates Oxidative Stress Injury and Inflammatory Infiltration in UUO Mice

**Figure 3:**
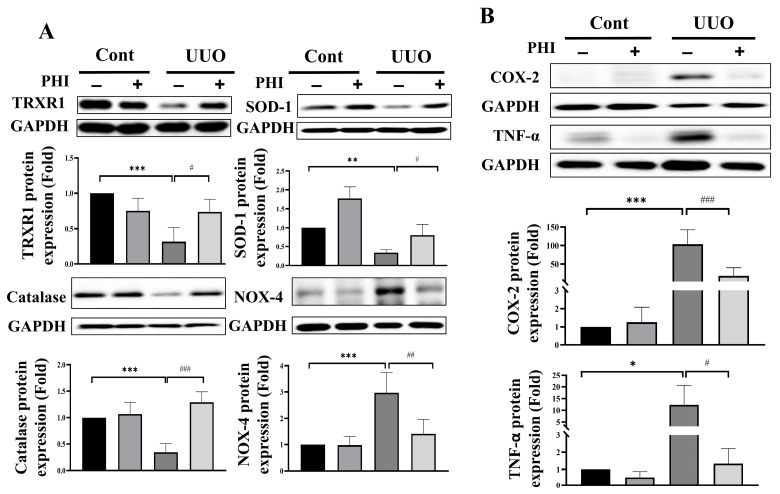
PHI attenuates protein expression of oxidative stress and inflammatory markers in UUO kidneys. Protein expression levels were analyzed by Western blotting. Expression levels of oxidative stress–related proteins, including TRXR1, SOD-1, catalase, and NOX-4. (**A**) Expression levels of inflammatory proteins, including TNF-α and COX-2. (**B**) Data are presented as mean ± S.D. 95% confidence interval (*n* = 6). * indicates *p* < 0.05, ** indicates *p* < 0.01, *** indicates *p* ≤ 0.001 when data are compared to the Sham Control group. # indicates *p* < 0.05, ## indicates *p* < 0.01, ### indicates *p* ≤ 0.001 when data are compared to the UUO group. Cont: Sham Control; PHI: Phillygenin.

**Figure 4:**
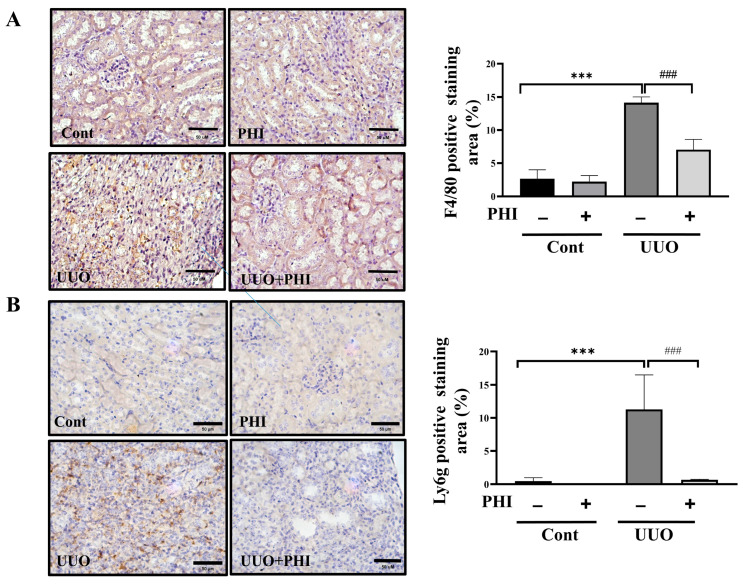
PHI reduces inflammatory cell infiltration in UUO kidneys. C57BL/6J mice were orally administered a vehicle, 25 mg/kg PHI, or 5 mg/kg Can for a consecutive 14 days after UUO surgery. Macrophages and neutrophil markers were detected by (**A**) F4/80 and (**B**) Ly6g, respectively. Magnification: 400×; Scale bar: 50 μm. Data are presented as mean ± S.D. with 95% confidence interval for each group (*n* = 6. In F4/80 detection, Cont: 3.241, 95% CI: 1.53 to 3.38; PHI: 2.84, 95% CI: 1.785 to 3.49; UUO: 14.78, 95% CI: 13.161 to 15.019; UUO + PHI: 7.217, 95% CI: 6.124 to 8.709; ANOVA: *p* = 2.62 × 10^−7^ Cont vs. UUO *p* = 1.6 × 10^−6^; UUO vs. UUO + PHI *p* = 2.33 × 10^−4^; in Ly6g detection, Cont: 0.702, 95% CI: 0.453 to 0.8.2; PHI: 0.1, 95% CI: −0.12 to 0.2; UUO: 12.24, 95% CI: 8.161 to 16.012; UUO + PHI: 1.202, 95% CI: 0.824 to 1.809; ANOVA: *p* = 2.41 × 10^−7^ Cont vs. UUO *p* = 1.6 × 10^−6^; UUO vs. UUO + PHI *p* = 2.01 × 10^−5^). *** indicates *p* ≤ 0.001 when data are compared to the Sham Control group. ### indicates *p* ≤ 0.001 when data are compared to the UUO group. Cont: Sham Control; PHI: Phillygenin.

Oxidative stress is a significant contributor to renal fibrosis, with the kidney—being an organ with high energy demands and abundant mitochondria—particularly susceptible to damage induced by reactive oxygen species (ROS) [4]. Next, we investigated the protective effect of PHI treatment on the protein expression of antioxidant enzymes, including thioredoxin reductase 1 (TRXR1), superoxide dismutase 1 (SOD-1), catalase, and NOX-4, in UUO mice. As shown in Figure 3A, the protein level of antioxidant enzymes, TRXR1, SOD-1, and catalase was significantly diminished, while NOX-4 was increased remarkably, in the UUO kidneys. After PHI administration, the level of antioxidant enzymes was significantly restored, and the level of NOX-4 was suppressed notably in UUO mice. Persistent inflammation has been identified as a crucial player in the formation of renal fibrosis and progressive CKD [10]. Interestingly, PHI has been reported for its potential anti-inflammatory effects in vitro and diseases such as colitis [16,33,34]. Next, we further evaluated the expression of inflammation-related proteins, including cyclooxygenase-2 (COX-2) and tumor necrosis factor-α (TNF-α), which were upregulated in the UUO kidneys and significantly contracted by PHI treatment. (Figure 3B) Additionally, PHI effectively decreased inflammatory cell infiltration, as evidenced by the reduced expression of macrophage marker (F4/80) and neutrophil marker (Ly6g) in the kidneys of UUO mice. (Figure 4) These results suggest that PHI administration possesses remarkable antioxidant and anti-inflammatory capabilities, potentially contributing to the amelioration of progressive CKD.

### 3.3. Administration of PHI Ameliorates Pyroptosis Injury in UUO Kidneys via Inhibition of the Activation of NLRP3 Inflammasome

**Figure 5:**
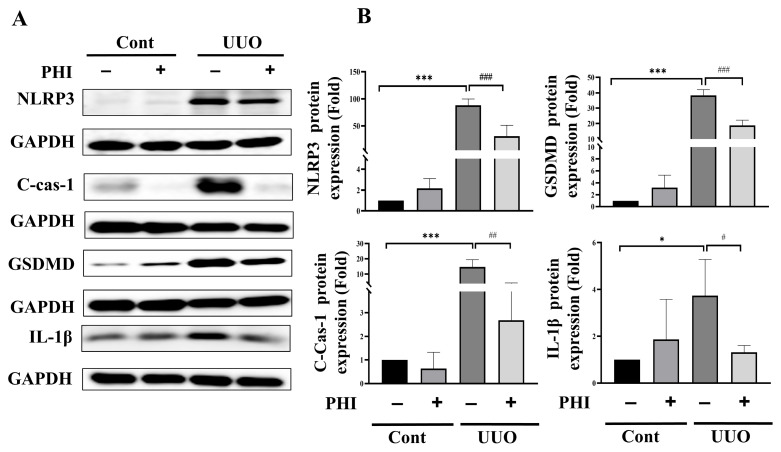
Administration of PHI suppresses pyroptosis in UUO kidneys via inhibiting the activation of NLRP3 inflammasome. Protein expression was analyzed by western blotting. (**A**) Quantification of protein levels is shown in (**B**). Data are presented as mean ± S.D. 95% confidence interval (*n* = 6). * indicates *p* < 0.05, *** indicates *p* ≤ 0.001 when data are compared to the contralateral control group. # indicates *p* < 0.05, ## indicates *p* < 0.01, ### indicates *p* ≤ 0.001 when data are compared to the UUO group. Cont: Sham Control; PHI: Phillygenin.

A recent study has indicated that pyroptosis, a novel type of inflammation-associated programmed necrotic cell death, plays a contributory role in CKD [12]. This process correlated with a strong inflammatory response by increasing expression of the NLRP3 and caspase-1 while enhancing the production of interleukin(IL)-1β and IL-18 in UUO mice [10,35]. Next, we investigated whether PHI treatment could mitigate pyroptosis in UUO kidneys. As depicted in Figure 5, the level of pyroptosis-related proteins, including NLRP3, Caspase-1, GSDMD, and IL-1β, was increased significantly, which PHI treatment successfully attenuated. These findings suggested that PHI treatment has therapeutic potential by alleviating pyroptosis and inflammation in UUO kidneys.

### 3.4. PHI Treatment Reduces Apoptotic Renal Cell Death in the Kidneys of UUO Mice

**Figure 6:**
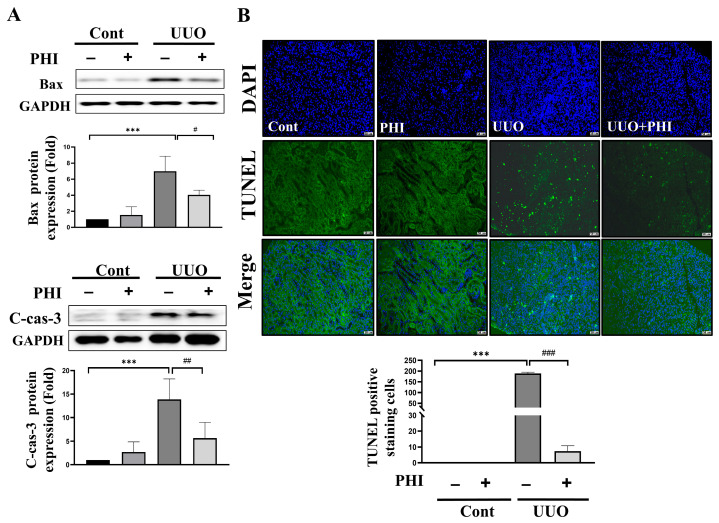
Administration of PHI reduces apoptotic renal cell death in UUO mice. Expression of apoptosis-related proteins (Bax and cleaved caspase-3) analyzed by Western blotting. (**A**) Apoptotic cells were assessed by Fluorescent TUNEL staining, and the quantification of positive cells was presented as mean ± S.D. 95% confidence interval for each group (*n* = 6). (**B**) *** indicates *p* ≤ 0.001 when data are compared to the Sham Control group. # indicates *p* < 0.05, ## indicates *p* < 0.01, ### indicates *p* ≤ 0.001 when data are compared to the UUO group. Cont: Sham Control; PHI: Phillygenin.

Increasing programmed cell death has been linked to cell infiltration, tubular atrophy, and interstitial fibrosis [36,37]. Next, we examined the expression of apoptosis regulators, such as Bax and caspase-3. As shown in Figure 6A, the protein expression of Bax and cleaved-caspase-3 was significantly upregulated in UUO kidneys, while PHI treatment reversed the increased death protein levels. Additionally, TUNEL-positive staining cells were markedly increased in UUO kidneys, a trend significantly mitigated following PHI administration (Figure 6B). These results suggest that PHI treatment can attenuate programmed apoptotic and necrotic cell death in UUO kidneys.

### 3.5. PHI Treatment Inhibitory Regulates ATP and LPS-Induced p-p65/NLRP3/GSDMD/IL-1β Pyroptosis Axis in NRK52E Treated

**Figure 7:**
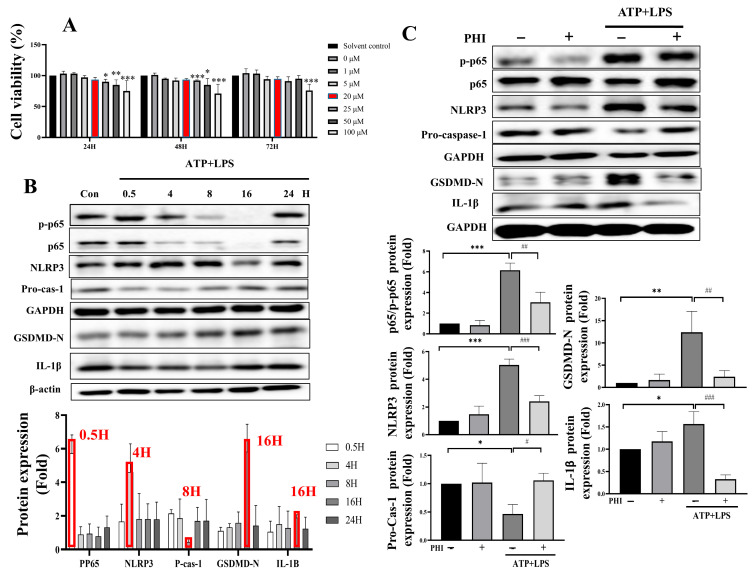
PHI inhibits ATP/LPS-induced pyroptosis signaling in NRK52E cells. Cell viability assessed by MTT assay after PHI treatment at different concentrations. (**A**) Time-course analysis of pyroptosis-related protein expression induced by ATP and LPS. (**B**) PHI treatment attenuated ATP/LPS-induced activation of pyroptosis-related proteins, including p-p65, NLRP3, caspase-1, GSDMD, and IL-1β. (**C**) Data are presented as mean ± S.E.M (*n* = 3). * indicates *p* < 0.05, ** indicates *p* < 0.01, *** indicates *p* ≤ 0.001 when data are compared to control group. # indicates *p* < 0.05, ## indicates *p* < 0.01, ### indicates *p* ≤ 0.001 when data are compared to the ATP + LPS group. PHI: Phillygenin.

To elucidate the involvement of the pyroptosis signaling axis, ATP and LPS treatment were used to simulate pyroptosis injury in NRK52E cells, as previously studied [38,39]. As shown in Figure 7A, cell viability was assessed by MTT analysis. Except for the concentration of 100 μM, there were no significant changes observed when compared to the solvent control. Based on other studies [40], 20 μM was selected for the subsequent experiments. Next, protein expression of pyroptotic-related markers, including pp65, NLRP3, caspase-1, GSDMD, and IL-1β, was elevated by ATP-LPS in a time course-dependent manner (Figure 7B). The highest protein expression level was selected for further analysis at each time point. As shown in Figure 7C, ATP-LPS treatment significantly increased the expression of pyroptosis markers, while PHI treatment notably alleviated the increased pyroptosis markers. These results suggest that PHI plays an important role in protecting against pyroptosis induced by ATP-LPS treatment in NRK52E.

A schematic illustration showing the proposed protective mechanism of PHI in UUO-induced renal injury and ATP/LPS-stimulated renal tubular epithelial cells. PHI suppresses activation of the NF-κB/NLRP3/caspase-1/GSDMD signaling pathway, thereby reducing pyroptosis, inflammation, oxidative stress, and renal fibrosis. These findings suggest that PHI may represent a potential therapeutic and nutraceutical candidate for mitigating CKD progression.

## 4. Discussion

In the present study, we demonstrated that Phillygenin (PHI), a plant-derived lignan isolated from *Forsythia suspensa*, exerts significant protective effects against renal injury and fibrosis in a unilateral ureteral obstruction (UUO) mouse model. Our findings show that PHI administration markedly attenuated renal fibrosis, inflammatory cell infiltration, oxidative stress, and pyroptosis-related signaling in UUO kidneys. Mechanistically, PHI suppressed activation of the NF-κB/NLRP3/caspase-1/GSDMD signaling pathway, which plays a central role in inflammation-driven pyroptotic cell death. In addition, PHI significantly inhibited ATP/LPS-induced pyroptosis in NRK-52E renal tubular epithelial cells. To the best of our knowledge, this study is the first to demonstrate that PHI alleviates renal fibrosis and CKD progression through inhibition of pyroptosis-associated signaling pathways. Given the increasing interest in plant-derived bioactive compounds as potential nutraceutical strategies for chronic metabolic and inflammatory diseases, our findings highlight the therapeutic potential of PHI as a promising candidate for mitigating CKD progression.

For dose selection, a previous study indicated that PHI treatment with 100 mg/kg per day for 21 consecutive days significantly inhibits apoptosis and fibrosis in chronic colitis mice without accompanying complications [18]. Another study found that oral administration of PHI of 50 mg/kg per day for 13 continuous weeks effectively ameliorates hepatocyte inflammation and hepatic cirrhosis in mice with fatty liver disease [41]. Furthermore, a recent study also identified a protective effect against persistent inflammation and fibrosis in diabetic mice with an intragastric administration of PHI (50 mg/kg/day) for consecutive 8 weeks [42]. Our study further selected a lower and safety dose to demonstrate that PHI treatment at 25 mg/kg per day for 14 continuous days relieves the pathological alterations, inflammatory cell infiltration, and necrotic cell death (pyroptosis) in mice with progressive kidney injury, suggesting that a prolonged treatment of 25–100 mg/kg PHI possesses a beneficial potential for the chronic diseases such as nonalcoholic fatty liver disease, chronic colitis, diabetes, and CKD in rodent models.

Fibrosis is a critical hallmark in progressive CKD [43]. Recently, studies have shown that PHI exhibits anti-fibrotic effects on hepatic stellate cell activation and inflammation [44], and pulmonary injury in mice via TGF-β dependent signaling [19]. Our results also demonstrated that administration of PHI inhibited the expression in the kidneys of UUO mice (Figure 3) and relieved TGF-β-induced protein markers of fibrosis, such as αSMA and fibronectin, in renal tubular cells in our non-public results. These results suggested that PHI possesses anti-fibrotic properties through the inhibition of TGF-β-dependent pathways, offering protection against chronic diseases such as CKD.

Pyroptosis, a subtype of programmed necrotic cell death, can cause direct multicellular organism or cellular damage, through the recruitment of acute inflammatory cells, and stimulation of inflammatory responses, which is a crucial process of kidney injury [12,45]. A recent study indicated that inhibiting pyroptosis through the knockdown of the associated factor, KLF4, prevents the initiation of tubular cell pyroptosis, inflammation, and renal fibrosis [46]. Wu et al. recently demonstrated that deletion of a pyroptosis-associated factor, Gasdermin E, attenuates renal fibrosis and kidney dysfunction in a 5/6 nephrectomy and a UUO mouse model. Similarly, our study also found that inhibition of pyroptosis networks via PHI administration contributes to the improvement of renal fibrosis, inflammation response, and further kidney injury in UUO mice. These results suggested that inhibition of pyroptosis could serve as a central target to prevent progressive renal injury in further studies. Interestingly, the nuclear transcription factor κB (NF-κB), a family of inducible transcription factors that drive inflammatory responses by modulating the production and release of cytokines [47], has been demonstrated to be involved in the canonical pathway of pyroptosis, enhancing the transcription of the NLRP3 gene, and further stimulates the maturation and secretion of pro-inflammatory cytokines such as IL-1β and IL-18 [10,47,48]. A recent study has shown that PHI treatment inhibits inflammation and apoptotic death by regulating NF-kb-dependent pathways in diabetic mice [42]. Another study demonstrated that administration of PHI attenuated colon inflammation and improved intestinal mucosal barrier in DSS-induced colitis via NF-kB and TLR4/Src signaling pathways in mice [49]. Zhou et al. have reported that PHI has a strong affinity for inhibition of the NF-κB pathway via molecular docking detection in an LPS-induced inflammatory response of a cell model. Our results also found that PHI treatment dramatically inhibits the expression of NF-kB in the early stage of pyroptosis networks, suggesting the inhibition of NF-kB may be an important target of PHI treatment in the early stages of pyroptosis.

The findings of this study have to be interpreted with consideration of certain limitations. First, while PHI treatment has been shown to improve pyroptosis signaling networks and further reduce fibrosis in UUO mice, the specific targets linking pyroptosis and fibrosis warrant further exploration using alternative cell models. Second, although we demonstrated significant inhibitory regulation of the pyroptosis axis, including NF-κB/NLRP3/Caspase-1/GSDMD, by PHI treatment, other non-canonical pathways in UUO mice and ATP/LPS-stimulated cells remain unidentified and should be further elucidated.

## 5. Conclusions

**Figure 8:**
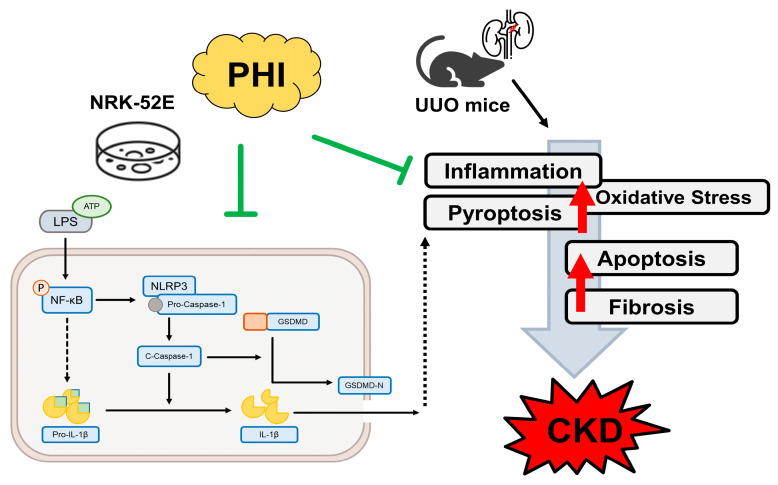
A schematic representation of the proposed signaling networks improved by PHI treatment in vitro and in a UUO mouse model. PHI treatment exerts inhibitory effects on inflammatory and cell death pathways in both in vitro (NRK-52E cells) and in vivo (UUO mouse model) systems. In NRK-52E cells, LPS stimulation activates the NF-κB signaling cascade, leading to NLRP3 inflammasome assembly (NLRP3, pro-caspase-1) and subsequent cleavage of caspase-1. Activated caspase-1 promotes the maturation of pro-IL-1β into IL-1β and induces cleavage of GSDMD to its active N-terminal fragment (GSDMD-N), triggering pyroptosis. In UUO mice, inflammatory signaling is associated with increased oxidative stress, apoptosis, and fibrosis, ultimately contributing to CKD progression. PHI attenuates these pathological processes. Green with blunt ends (⊣): indicate inhibitory effects of PHI on signaling pathways. Black arrows (→): represent activation or progression of molecular events. Red arrows (↑): denote upregulation of pathological processes. Dashed arrows: indicate indirect effects.

In conclusion, our findings provide a novel perspective on the amelioration of progressive CKD through PHI treatment in both a UUO mouse model and in vitro. We demonstrated that PHI significantly alleviates renal fibrosis, inflammation, and pyroptosis in a UUO mouse model. In addition, PHI suppresses ATP/LPS-induced pyroptosis in renal tubular epithelial cells via inhibitory regulation of the NF-κB/NLRP3/caspase-1/GSDMD signaling pathway, as illustrated in the schematic diagram in Figure 8. As a lignan-derived phytochemical isolated from *Forsythia suspensa*, PHI represents a promising plant-derived bioactive compound with potential nutraceutical value. These findings highlight the therapeutic potential of PHI for the prevention or attenuation of CKD progression.

## Data Availability

The complete dataset used in this study will be made available to researchers upon reasonable request from the corresponding authors.

## Abbreviations

The following abbreviations are used in this manuscript:

ATP	Adenosine Triphosphate
CKD	Chronic Kidney Disease
CTGF	Connective Tissue Growth Factor
ECM	Extracellular Matrix
EMT	Epithelial–Mesenchymal Transition;
GSDMD	Gasdermin D
IL-1β	Interleukin 1 Beta
LPS	Lipopolysaccharide
NF-κB	Nuclear Factor kappa B
NLRP3	NLR Family Pyrin Domain Containing 3
NOX	NADPH Oxidase
PHI	Phillygenin
TRXR1	Thioredoxin Reductase 1
UUO	Unilateral Ureteral Obstruction
